# Mechanical Dentistry

**Published:** 1876-12

**Authors:** J. Curtiss Smithe


### MECHANICAL DENTISTRY.

BY DR. J. CURTISS SMITHE.

*Read before the Maryland State Dental Society.*

Mr. President and Gentlemen:—Your committee appointed to report on mechanical dentistry, beg leave to offer for your consideration, acceptance, or rejection, the following report:

It is the opinion of a large number of dentists that mechanical and operative dentistry should be separate and distinct. Your committee take issue with these gentlemen, and will present as concisely as possible their reasons for so doing: 1st. It is generally conceded that dentistry as an art partakes largely of mechanical appliances, and he succeeds best in the operative department who by his mechanical skill brings his manipulative power to harmonize. The brain may conceive an idea, but without manipulative mechanical ability to carry this out, it amounts to naught. If the majority of our profession have passed through their primary education and become adepts in their art, it may be well and proper enough for even those to have what is called a supervision of mechanical dentistry in the mouth. By that is meant the proper adjustment of the teeth in relation to contour, features, the proper distention of relaxed muscles, and general harmony of case when completed. To be able to do this successfully, your committee conceive it to be equally important and necessary that the mechanical should receive just as much attention and close study as the operative branch, and this harmonizing of the two makes what is known and recognized, both by the community at large and our profession, the most successful practitioner of modern dentistry. Your committee would not have the profession misunderstand them in this respect. They do not for a moment oppose the custom of a large number in full practice of having their mechanical work done by assistants, or by those who do the mere mechanical drudgery, but they do contend because a gentleman finishes a case that to all appearances looks well in the hand, it is no criterion to judge of his ability to successfully grapple with the various and varied mechanical appliances that certain conditions of the mouth render at times absolutely necessary. In this connection we ask your consideration to a quotation from Mr. H. Sewell's letter in last "Cosmos:" "To practice this art safely, a professional education is required, such as is undergone by candidates for license in dental surgery of college of surgeons. This course of study, besides practical instruction in dental surgery and mechanics, provides that the candidate shall go through a course of instruction in general anatomy and surgery, so that, having an acquaintance with the nature of disease and the principles of medical practice, he may avoid error and be able to deal with confidence with the special region of the body to which his treatment will be confined. Not to speak of the mechanical treatment of cleft palate, of deformities of the jaws, of irregularities of the teeth, we may safely affirm that every dental surgeon meets almost daily with cases which could have presented originally no difficulty, but which, having been treated by uneducated mechanics, without regard to the anatomy and physiology of the teeth and parts influenced by the apparatus, have resulted in the infliction of unnecessary, intense, and prolonged suffering."

Gentlemen, how can you separate one from the other in a compound fracture of either superior or inferior maxillary? The combination of both operative and mechanical ability is so closely allied in this and kindred operations of congenital or syphilitic cleft palate that a separation means disaster and failure.

If the gentlemen who are contending for the separation of the specialties are sincere, let them place upon their cards (as some do) "operative dentist," and let them adhere to the strict understanding of that word, that they only take charge of the natural teeth and diseases incidental thereto, and let those gentlemen who write dentist after their name be fully prepared by a proper education to take charge not only of the natural teeth and diseases incidental thereto, but the difficult cases arising from fracture of jaws, correction of irregularities, making artificial obturators and artificial teeth when all other means have failed to preserve the natural ones intact.

Your committee felt it their duty to call the attention of this meeting to the fact that there is, as a rule, too little preparation in the mechanical branch of our profession, and as a consequence irresponsible parties have taken up and thrown their bad odor upon the more respectable ones in the profession, from the fact that the vegetable bases are taught almost to the exclusion of metallic. Students even in dentistry are taught this, and in some cases their education ceases at this point. They become accustomed to this style of work, from the fact that they know no other. This is not as it should be. They should be instructed in all kind of bases known at the present time. Then they will be competent to judge of the merits and demerits of each and select understandingly. Gentlemen argue that the people demand this cheap style of work. If so, why? Are not our professional brethren to blame? Are they not the instructors of their patients?

The subject in which we all should be interested, is the defects in artificial teeth as they are sent to us from the manufacturers, We think they are capable of improvement, especially in arrangement and proportion. One marked defect is in the too frequent separations, which is carried to excess. Take for instance a block of two incisors and a cuspid, with the lateral overlapping the central. Do you find separations in such cases in the natural teeth? Irregularities of this description are usually produced by want of room, how could there be a space? In a perfect set of teeth you find very few separations unless produced by a file in the hands of a dentist, and that to cure a disease, which of course is a deformity. The difficulty is in too much mechanical and too little of the artistical. We need separations sometimes; for instance, where we are to match natural teeth that have been separated. This universal separation, however, of all classes and kinds is a sad mistake. Another difficulty is the cuspids; they are, as manufactured, characterless. They are not wide enough, and have not the prominence that they have in the arrangement of the natural teeth. If any one will take the trouble to measure, he will find in a majority of his patients' mouths, unless attacked by disease, the cuspid is as wide as the central incisors; you will not find that the case with a majority of the cuspids on artificial blocks; besides the fact that there is not usually prominence enough along the center of the labial surface. It is also the fact that the gums above them should have more prominence than over the incisors. For expression, we are somewhat dependent on these same cuspids, and there has always been a very great difficulty in restoring the features at this point. We are aware that it is impossible to carry an artificial gum or plate as high as the root of a cuspid extends; there are many losses we can not fully restore; though we can more perfectly restore the features at points where we can get at them, provided we can get the teeth to do it with.

The bicuspids and molars are more frequently too small than too large. This is especially the case with bicuspids; they are often so made that it is impossible to carry them in as far as desirable, without reducing them so much by fitting that they frequently come from vulcanization a ruin. By observation you will find that with few exceptions the cuspids are the object teeth, and the bicuspids run almost in a direct line back from them. If you attempt to bring this about with with the present artificial bicuspids, you will find that you have cut into the pin-heads of both blocks, thereby weakening their hold.

Still another criticism deserving attention: a majority of the teeth are too young in expression. We are not so frequently called upon to insert teeth for the young people as our predecessors, (thanks to the advancement of our profession,) but for the middle aged and the aged. This youthful expression is partly done by shaping the cutting edges, and more by carrying the gum to a point between teeth. We can remedy the cutting edge by grinding, but we can not remedy the gum defect. You will usually find that in the middle aged the gums end more abruptly than in youth, and that there is a tendency to recession at those points. It is our duty to correct in so far as we can the deformities produced by the loss of the natural organs. This loss is a great misfortune, and our greatest efforts, no matter how skillfully and artistically applied, fail to make the waste places perfect; therefore it behooves us to do our best, not only to do our part but to use our influence with the teeth manufacturers to have them improve upon their already improved patterns.

Your committee present for your consideration a material known as "White's" modeling compound for impressions, as a valuable preparation, and much superior to wax for sharp outlining. Would also call your attention to a method of taking impressions in gutta-percha, known as the "Fouke" method, as novel, original, and well worthy your attention. It consists of an impression cup with the palatine portion removed; across this bands are placed of some yielding material such as canvass, so the gutta-percha can be forced into all the undercuts; it is then removed, chilled, and replaced a sufficient number of times to prevent its contraction. These two are the only new improvements in either material or method known to your committee. On materials for bases for artificial teeth, but one new discovery has been reported, that of a base prepared from *asclepias cornuti*, the gum of the common milk weed, in which experiments are being made; but so little is known of it, that we are not prepared to report upon its merits.

This report, if in harmony with the inclination of the writers, would be silent on "Vulcanite." If used, they would recommend as the strongest and best preparations, White's brown, G. D. V. Co's red, Ash & Son's brown, and Cole's red and brown. The pink and light rubbers are loaded heavily with foreign matters, such as white clay and oxide of zinc, and consequently are less strong. It has some advantages in the ease of adaptation over the other vegetable bases, but is much more liable to produce inflammation. If more time at a lower temperature was taken in the vulcanization of rubber, better results would be obtained, the difficulty of porosity would be gotten rid of, and more elasticity of plate gained. The methods as introduced by Stuck & Co. some seven years ago, of hardening the plate between sheets of tin, have some advantage over the ordinary way of manipulation, and returns for labor expended, a more satisfactory result.

There are but two varieties of collodion bases at present deemed of sufficient importance to claim the attention of the profession—rose-pearl and celluloid. The introduction of these materials was hailed with delight by the profession at large as a means of escape from the unjust taxation of the rubber monopoly; and not alone for this, for the better and more thoughtful men of our specialty have long felt the need of a better material as a base plate, and we are fearful this very anxiety has caused in a measure the placing of these materials upon the market before they were brought to a proper state of perfection by the manufacturers, and results have been less satisfactory from this cause, perhaps, than any other. We know there has been great improvement in this respect; the plates offered of late have been of better color, more uniform in texture, and, by the substitution of hemp for cotton, more strength is claimed. We have been speaking of these materials—rose-pearl and celluloid—as having the same base; that they have is undoubted, yet there is a marked difference in the methods of manipulation—one, rose-pearl, requiring (owing to the greater hardness of the material) a number of metallic castings, upon which it is moulded. That more time is necessary in this process is evident. The additional time, however, would be no objection if better results were obtained than by its kindred material, celluloid. With present information, we are not prepared to report in the affirmative, neither can we deny the claim of the originators that this is so. Of the two, celluloid has claimed the larger share of attention from the profession, probably owing to the fact that apparently the same result is reached in less time, with less labor, and with an equal degree of certainity.

We would recommend these materials to the careful consideration of the profession. That they are stronger and lighter than rubber is an undeniable fact. Objections have been made of their liability to warp under certain conditions. That this has been the case is also undeniable. That a vast improvement has been made in this respect, owing as is claimed, to a more thorough tempering of the plates, none can doubt. That this objection, also other imperfections, with enlarged experience and facilities of the manfacturers, may be entirely overcome, is both possible and probable.

We would go more fully into detail of this material were it not that a gentleman present, Dr. Hunt, has given this subject his close attention and is better qualified to report upon its merits and demerits than any gentleman known to your committee at the present time.

Of the metallic bases, silver is probably the most objectionable, owing, first, to the great readiness with which it is oxydized. Certain conditions of the saliva produce an unpleasant metallic taste, and also corrode the plate to such an extent as to render it useless. Used only as a temporary substitute.

Aluminum has been used to a limited extent, and with no very satisfactory results. The casting process—that is, casting the plate as an attachment to the teeth—has proved a failure. A method of striking up the plate, tinning on little loops, and attaching the artificial teeth by rubber is recommended by some. This makes a light and beautiful case when finished, and in some mouths proves very satisfactory; in others, owing to the condition of the secretions, it will become pitted and roughened.

Weston's alloy makes, when cast properly, a very substantial lower case. It is liable to oxydization, and changes color slightly in the mouth. To prevent this it is recommended that all cases be electroplated with gold,

The other cheoplastic alloys are unequal, and not used to any extent.

We will call your attention but to two other bases, which deserve, we think, especial mention, from the fact that they have stood the greatest test—the test of time. We allude to gold and platinum.

The method of mounting sectional blocks to a gold base by the aid of rubber or celluloid is by some used to a considerable extent, and makes a very pretty, strong and cleanly case. The only precaution necessary is to have the loops so adjusted as not to interfere with the blocks when brought into position.

Carved blocks admit of a greater amount of artistic skill. They can be carved in the most irregular and natural shapes, approaching more nearly nature's handiwork, perhaps, than any other method known (continuous gum alone excepted). When blocks are used it is much better to have a continuous band extending from the lingual to the labial portions of the plate. This style of mounting is pretty, strong, and cleanly, and will fully compensate for additional labor. The objection urged by some, that in soldering the blocks or single teeth to plate causes it (the plate) to warp, is not an objection well founded, when sufficient care has been taken in the preliminary operation of embedding the case. This claimed objection, however, is entirely overcome by the method previously mentioned.

Platinum base, continuous gum, or "Allen's work," as it is oftentimes called, (out of compliment to the original inventor,) is a method too little used by the profession. It is beyond doubt applicable in a greater number of difficult cases than any other method at present known. It is cleanly, strong, beautiful, and admits of an amount of artistic skill almost unlimited. The only objections urged to this work are, 1st, its weight; 2d, the time necessary in its preparation.

That it is frequently made unnecessarily heavy by inexperienced manipulators is undeniable. That this need not be the case is fully proven by the large number of cases satisfactorily worn by patients at the present time. It is not easily broken, and admits of being easily repaired.

If time in its preparation be an objection, when adequate results are obtained, your committee would do injustice to the intelligence of this convention by discussing.

Gentlemen, the millenium has not yet arrived. Artificial teeth will have to be inserted through this generation at least. The best men in the profession must step forward and place this branch of our specialty on an equality with the operative—lift it from the mire of indifference and carelessness that has characterized it for the past few years; give to it the proper attention it so richly merits; instruct your patients in the better methods, and demand from them remunerative fees. In this you hasten a reaction so long needed, and at the same time rebuke those who have merely "picked it up," and are loudest in mouthing their own achievements.

Those who study closely the expressions upon the face divine and correspondingly work out results in harmony, to those thanks, hearty thanks, are due. To just such honest, fair minded men are we indebted for our standing and success.

To those who have for their motto, cheapness, not quality, let us anticipate a few years public opinion and commit them to the oblivion their actions merit.

